# Experimental study on mandibular length and facial symmetry of low estrogen level and anterior disc displacement of temporomandibular joint

**DOI:** 10.1038/s41598-018-34023-4

**Published:** 2018-10-23

**Authors:** Zixian Jiao, Xiangyu Wang, Xiaohu Zhang, Chi Yang

**Affiliations:** Department of Oral Surgery, Shanghai Ninth People’s Hospital, Shanghai Jiaotong University School of Medicine, Shanghai, 200011 China

## Abstract

This study was aimed at elucidating the changes of mandible symmetry in a rabbit model with low estrogen levels induced by ovariectomy (OVX) combined with temporomandibular joint (TMJ) anterior disc displacement without reduction (ADDWoR). 32 growing rabbits were randomly allocated into 4 groups; OVX group, ADDWoR group, OVX+ ADDWoR group and control group. In OVX and OVX+ ADDWoR groups, bilateral OVX was performed and then the serum level of 17β-estradiol was evaluated every week. In ADDWoR group and OVX+ ADDWoR group, the right TMJ was surgically opened and the disc was displaced anteriorly and the left TMJ was also surgically opened and closed without any manipulation of the disc. All rabbits had CT scan before and at the end of the study and the mandible measurements were performed on the 3D-reconstructed model. The mandible in ADDWoR group was consistently shorter on the right side resulting in a midline shift to the ipsilateral side. While in OVX+ ADDWoR group, the mandibular length of the right side was more shorter than in ADDWoR group, moreover, mandibular deviation was therefore more severe. In OVX group, there was no difference regarding the length of mandible compared to the control group. There was no difference regarding the mandibular length between left and right sides in the control group.

## Introduction

Anterior disc displacement (ADD) of the temporomandibular joint (TMJ) is one of the most common disorders of the maxillofacial region with a high prevalence in female adolescents. Based on different pathological changes, ADD can be divided into two types: ADD with reduction (ADDWR) and ADD without reduction (ADDWoR). It has been previously reported that unilateral ADDWoR in teenagers will lead to asymmetry of the condyle and mandible^1^ and with the prolongation of the history, the mandible asymmetry will become more severe^2^. The displaced TMJ disc might be reducing at an earlier stage, then progresses to non-reducing form later. TMJ DD is associated with altered condylar morphologies, such as decreased condylar height and distally inclined condyles. The altered condylar morphologies become more severe as TMJ DD progresses to nonreduction. Both qualitative and quantitative condylar changes are associated with TMJ DD. Osseous changes of the mandibular condyle is significantly influenced by TMJ DD and that altered condylar morphologies become more severe as TMJ DD progresses^3^. Furthermore, when ADDWoR is further developed, it will cause severe bone resorption, known as idiopathic condylar resorption (ICR). ICR is a well-documented however still unclearly identified disease. The typical clinical manifestations of ICR include mandibular retrusion, class II occlusal relationship, open bite of the anterior teeth, in addition to ADDWoR accompanied with a very small condyle evident on an MRI examination. ICR is a disease entity associated with a variety of factors including local factors, which produce compression such as internal derangement (ID) and systesmic factors such as systemic arthritis and hyperparathyroidism. Due to the high occurrence of ICR in female adolescent patients, many scholars have assumed that the sex hormones might be related to ICR^4^. Evaluation of serum estrogen levels in ICR patients revealed a significant reduction of the 17β-estradiol level^5^. Whether it is the ADD or low serum estrogen level that plays a more important role in the progression of ICR remains unclear and controversial. Thus, this study aimed at verifying the influence of a low serum estrogen level and ADDWoR on mandible length and asymmetry and discussing the probable initial cause of ICR.

## Results

### Changes in rabbit body weight throughout the experiment

**Figure 1:**
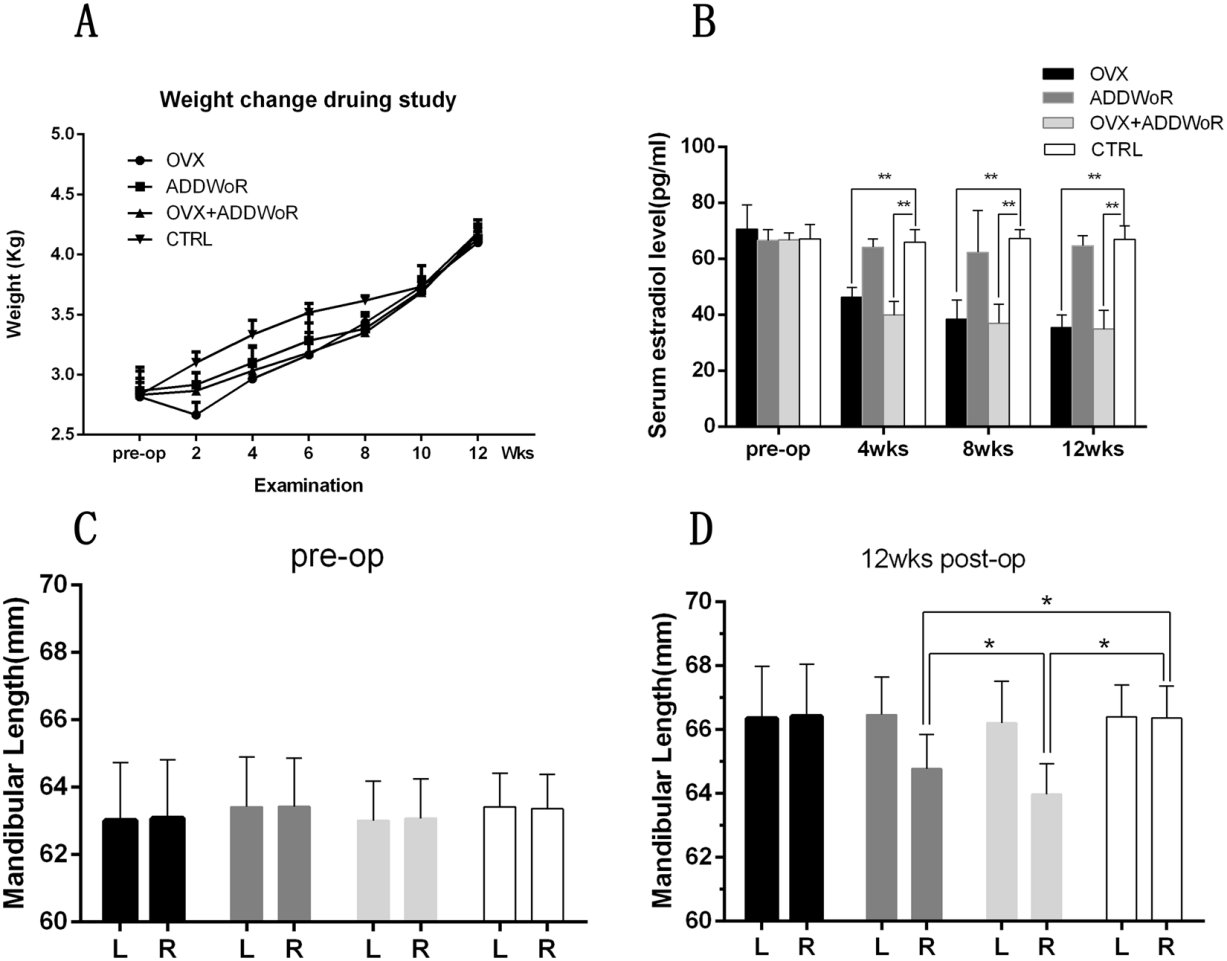
The changes of rabbits’ body weight, serum estrogen level and mandible development throughout the study. (**A**) There was no significant difference of body weight between groups before and at the end of the study. (**B**) There was no significant difference of the serum estradiol level between groups at the beginning of the study. After OVX surgery, serum estradiol level in OVX and OVX+ ADDWoR group was significantly reduced compared to control group (*p < 0.05). No difference was found between ADDWoR group and control group. (**C**) No difference was found between groups of mandibular length before surgery. (**D**) There was significant difference (*p < 0.05) of mandibular length of right side between ADDWoR group and control group, and the mandibular length of right side in OVX+ ADDWoR group was significantly shorter than that of ADDWoR group.

The average body weight of 32 female New Zealand white rabbits before surgery was 2.84Kg (ranging from 2.5 to 3.2 Kg). As shown in Fig. 1A, statistical analysis was performed after randomization and no significant difference was found between the four groups (*p* > 0.05). 2 weeks postoperatively, the body weight of OVX group decreased slightly and then increased gradually till the end of the study, with the mean body weight increasing from 2.55 Kg (2 weeks) to 4.1 Kg (12 weeks). In the ADDWoR group and OVX+ ADDWoR group, almost no change of the body weight was found in the first 2 weeks, however, the mean body weight increased from 2.72 Kg (2 weeks) to 4.13 Kg (12 weeks) and 2.75 Kg (2 weeks) to 4.16 Kg (12 weeks), respectively. In the control group, the body weight increased steadily from 2.83 Kg (2 weeks) to 4.18 Kg (12 weeks). By the tenth week and beyond, there was no significant difference in body weight between the four groups (*p* > 0.05). No animal was lost during the study period.

### Serum 17β-estradiol assay

To verify whether the OVX model was created successfully, the serum 17-β-estradiol level was assessed at intervals. As shown in Fig. 1B, no significant difference was observed among the 4 groups before the operation of OVX. In OVX group and OVX+ ADDWoR group, the serum 17-β-estradiol level was significantly reduced (*p* < 0.01) from the 4^th^ week till the end of the study, which means the model of OVX was successfully established. While in ADDWoR group, the 17 β-estradiol maintained at a steady level with no significant difference compared to the control group.

### Mandibular length

**Figure 2:**
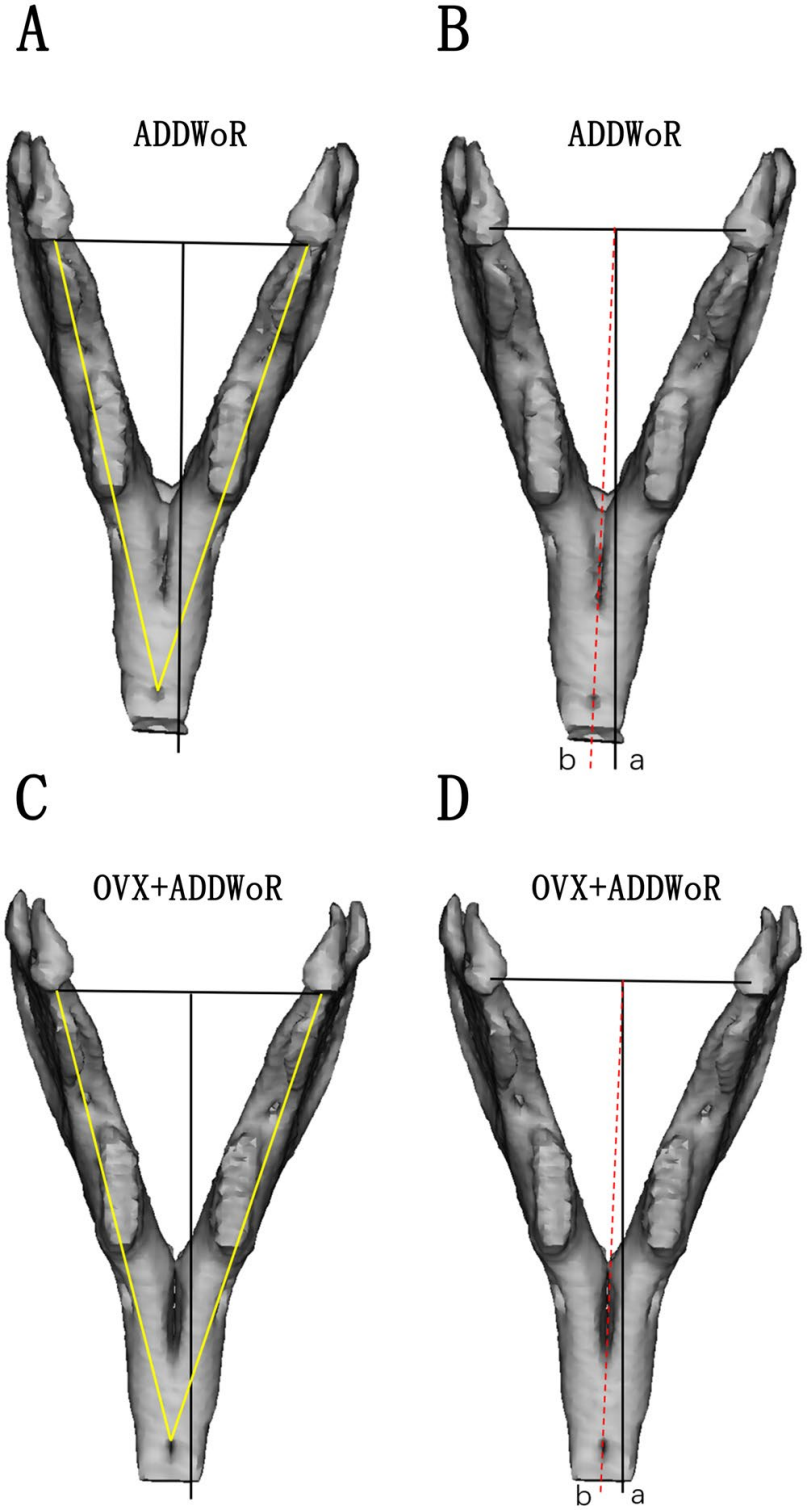
The mandibular length and midline asymmetry of the operated group. The mandibular length was considered as the distance from the end of jaw bone between the incisors to the anterior border of the condyle. The angle between line a and line b was identified as midline shift. (**A**,**C**) In ADDWoR and OVX+ ADDWoR group, the length of right mandible was significantly shorter than the left side. The length of right mandible in OVX+ ADDWoR group was even shorter than that in the ADDWoR group (*p < 0.05). (**B**,**D**) Mandible asymmetry was found both in ADDWoR and OVX+ ADDWoR group, the angle between line a and line b in OVX+ ADDWoR group was larger than that in the ADDWoR group (*p < 0.05).

All mandibles were well reconstructed with a smooth surface and the measurements were made directly on the 3D model. The mandibular length of all animals was measured and the results revealed good mandibular symmetry before surgery with no significant difference (*p* > 0.05) in mandibular length between the left and right sides (Fig. 1C), making the change of mandibular length between groups comparable. In control group, no difference was found between the left and right sides with a mandibular length of 66.41 mm and 66.36 mm, respectively, 3 months postoperatively. In OVX group, the mandibular length increased evenly, and the measurements revealed good facial symmetry with no significant difference of mandibular length between both sides (*p* > 0.05) and no difference was found between OVX group and the control group. While in ADDWoR group (Fig. 2A) and OVX+ ADDWoR group (Fig. 2C), the mandibular length of the right side was significantly shorter (Fig. 1D) than the left side (*p* < 0.05), and in the above two groups, no difference was observed in the mandibular length of the left side compared to control group. The mandibular length of the right side of OVX+ ADDWoR group was significantly shorter (*p* < 0.05) than that of ADDWoR group (Fig. 1D).

### mandibular shift

The mandible in the control group and OVX group showed good symmetry with almost no midline shift. In ADDWoR and OVX+ ADDWoR group, the mandibular symphysis consistently shifted toward the operated side (Fig. 2B,D). A statistically significant difference was found between ADDWoR group and control group (*p* < 0.05). The angles in the OVX+ ADDWoR group were larger than that in the ADDWoR group.

## Discussion

Unilateral ADDWoR of the TMJ would impairs condylar height, leading to facial asymmetry, which was verified both clinically and experimentally. In adolescent patients, ADDWoR was found to be accompanied with a decreased condylar height^6,7^ and with the prolongation of ADDWoR history, the condylar height tended to decrease more^8^. In animal models, an experimentally induced unilateral ADDWoR of the TMJ would impair the ipsilateral condylar growth^9,10^. In the current study, the mandible growth of ADDWoR sides was also affected. In ADDWoR group, the mandibular length of right sides was significantly shorter than of the left sides, and the midline of the mandible was shifted towards the operated side, indicating that the ADDWoR animal model was built successfully. In OVX group, the mandible revealed good symmetry with no midline shift and no difference of mandibular length was found between OVX group and the control group. While in OVX+ ADDWoR group, the effects on mandibular growth were more severe, therefore, the mandibular length decreased more, and the midline was shifted more to the right side compared with ADDWoR group. The serum 17β-estradiol level in OVX group and OVX+ ADDWoR group was reduced by one-third, which mimicked the condition in young patients with a low serum estrogen level. Throughout this study, the animals’ body weight gained gradually, and no difference was found among all groups at the end of the experiments, which can exclude the effect of malnutrition on mandibular growth. In this study, there was no difference of mandibular length between OVX group and the control group, indicating that although OVX was verified to influence the condylar cartilage proliferation and turnover in mice, OVX does not affect the development of the mandible, including mandibular length and facial symmetry.

Thus, the hypothesis of this study was that low levels of serum estrogen alone will not affect mandibular growth, while low levels of estrogen together with ADDWoR, will lead to more severe facial asymmetry compared to ADDWoR alone.

Since the association of estrogen deficiency and osteoporosis was first described by Fuller Albright^11^, many studies has demonstrated that estrogen is the key regulator of bone metabolism^12^. In human body, osteocytes have the ability to sense the mechanical strain and then developing microcracks, in addition to the response to hormonal changes such as estrogen deficiency. Also, osteocytes represent an important source of receptor activator of NF-kappaB ligand (RANKL)^13^, which is the key molecule needed for osteoclast formation and function^14^. Estrogen has direct effects on osteoclasts such as decreasing the activation of NK-kappaB and impairing RANKL-induced osteoclastogenesis^15^ and suppressing RANKL-induced osteoclast differentiation^16,17^. Estrogen also induces osteoclast apoptosis, therefore, estrogen deficiency would lead to an increase in osteoclast lifespan due to reduced osteoclast apoptosis^18,19^. Through 3D-finite elements analysis, the pressure on the condylar surface increased after ADDWoR, especially the posterior surface of the condylar head^20^ and functional overloading, such as bruxism and prolonged clenching, may result in degenerative changes of the TMJ^21,22^. Thus, we have reasons to believe that the increased mechanical overloading following ADDWoR might lead to microcracks of the cancellous bone of the condyle, resulting in an excessive osteoclast formation. Under normal circumstances, osteoclasts are eliminated through an estrogen induced osteoclast apoptosis and only causing mild facial asymmetry. While under the circumstances of low serum estrogen level, the inhibitory effect of estrogen on osteoclastogenesis was attenuated, and estrogen induced osteoclast apoptosis was also decreased, leading to severe facial asymmetry compared to ADDWoR. Ongoing further studies are advocated to demonstrate the possible mechanisms of osteoclasts in the development of ICR.

## Materials and Methods

### Animals

32 3-month old female New Zealand rabbits were included in this study. These experiments were approved by the local ethics committee of Shanghai Jiao Tong University, School of Medicine and the guidelines for care and use of animals were followed. The rabbits were kept individually in cages and maintained on a “12-hour light/12-hour dark” cycle at room temperature with ad libitum access to water and a standard commercial rabbit chow.

After being acclimatized to the new conditions for 2 weeks, the rabbits were randomized into 4 groups: (1) Ovariectomy (OVX) group (n = 8): an experimental group in which OVX surgeries were performed combined with sham- ADDWoR operations of the TMJs. (2) ADDWoR group (n = 8): an experimental group in which ADDWoR of the TMJ was surgically created on the right side combined with sham-OVX operations. Sham- ADDWoR operations were performed on the left sides. (3) OVX+ ADDWoR group (n = 8): an experimental group in which ADDWoR of the TMJ was surgically created on the right side combined with the OVX surgery. sham- ADDWoR operations were performed on the left sides. (4) non-operated control group (n = 8). For the sham-OVX operation, the ovaries were held up and then returned to their original positions. For the sham- ADDWoR operation, the joint capsules were exposed without any manipulation of the TMJ disc.

All rabbits were 12 weeks old at the beginning of this study and were observed for 3 months, which is the rabbit’s growing period, equivalent to the adolescent in human^23^.

### Computed Tomography scanning

All animals had computed tomography (CT) scanning under general anesthesia at the beginning of the study and again after sacrifice. CT scans were obtained using a GE Discovery Elite PET-CT(USA), with the parameters of 80 Kv, 120 Ma, and a thickness of 0.625 mm. Then the DICOM files were copied and imported into the mimics 19.0 software (Materialise, Belgium) and the rabbits’ mandibles were 3D reconstructed for further analysis.

### Weight gain

All experimental animals were weighed and recorded preoperatively. During the study period, the body weight of each individual animal was recorded every 2 weeks to detect signs of malnutrition that may affect the growth of the animal.

### Surgery

#### TMJ surgery

Unilateral ADDWoR of the TMJ was created in each experimental animal as previously described^24^. Briefly, after general anesthesia, a 4-cm anteroposterior incision was performed on the right TMJ from the skin overlying zygomatic arch to bone contact. Then a hole was drilled about 40 mm from the root of the zygomatic arch and a stain-less wire was pierced through to fix the two elastic bands. Following the exposure of the TMJ capsule, the anterior part of the disc was sutured and the suture was knotted to the elastic bands which was stretched to 14 mm to create a tension force of 1 N^24^. When these procedures were completed, the wound was thoroughly irrigated by saline and closed in layers. The same procedure steps were performed for the ADDWoR sham-operated animals, except for the forward traction of the disc.

Antibiotics (penicillin, 0.1 g/kg/day, China) were administered 5 days after surgery to prevent postoperative infection of the tested animals.

#### OVX surgery

The OVX surgery was performed according to Pennypacker’s method^25^. Briefly, under general anesthesia, a 5-cm incision was made along the midline of the ventral abdomen extending caudally and ending 1 to 2 cm cranial to the pubis. The ovaries were exteriorized with caution, then the ovarian vessels and uterine tube were ligated and then the ovaries were removed. In the OVX sham-operated animals, the ovaries were exteriorized and returned again to its original position intact. The muscles and skin were sutured, and a light abdominal bandage was applied to protect the incision.

Antibiotics (penicillin, 0.1 g/kg/day, China) were administered 5 days after surgery to prevent postoperative infection.

#### Serum estrogen assay

At the beginning of this study, the serum 17β-estradiol levels were measured for all rabbits at baseline. During the experiment process, blood samples were collected from each individual experimental animal to assess the serum level of 17β-estradiol every 2 weeks. After centrifuge of the blood samples at 2000 × g for 20 minutes, 2 ml serum sample was extracted from each blood sample and stored at −80 °C for further analysis. 17β-estradiol assessment was performed using an enzyme linked immunosorbent assay(ELISA), following the manufacturer’s instructions (R&D System, Inc., Minneapolis, MN, USA)^26^.

#### Mandible analysis

Following CT scan, the rabbits’ DICOM files were imported into MIMICS 19.0 software (Materialise, Belgium) to reconstruct the 3D-model of the mandible. Thresholding was selected as min 326 and max 3071, then the mandible was separated from the skull and 3D-reconstructed. Each animal mandible was measured twice with an interval of 2 weeks.

#### mandibular length

The length of the mandible at each side was measured on the 3D model. The mandible length was considered as the distance from the end of the jaw bone between the lower incisors to the anterior border of the condyle^27^.

#### Midline shift

The rabbit’s facial symmetry was determined by the midline shift. The central point of each condyle was connected by a line and a perpendicular line was drawn from the midpoint to the frontal area of the mandible. Then another line was drawn from the midpoint of the inter-condyle line to the end of the jaw bone between the incisors. Then the angles between both lines were measured as illustrated in Fig. 2B ^9^.

#### Statistical analysis

All data were expressed as the mean ± SD. Results were analyzed with Analysis of Variance (ANOVA) using SPSS 13.0 software (SPSS Inc., USA). *P* < 0.05 indicated a significant difference between the groups.

#### Equipment and settings

Figures of statistical analysis including Fig. 1A–D were obtained using GraphPad Prism program version 6.01. Figure 2 showed mandibular growth and facial asymmetry, and CT scans were obtained using a GE Discovery Elite PET-CT(USA), with the parameters of 80Kv, 120 Ma, and a thickness 0.625 mm and 3D reconstructions were obtained using MIMICS 19.0 software (Materialise, Belgium).
